# Automatically rating trainee skill at a pediatric laparoscopic suturing task

**DOI:** 10.1007/s00464-017-5873-6

**Published:** 2017-10-25

**Authors:** Yousi A. Oquendo, Elijah W. Riddle, Dennis Hiller, Thane A. Blinman, Katherine J. Kuchenbecker

**Affiliations:** 1 Department of Mechanical Engineering & Applied Mechanics, University of Pennsylvania, Philadelphia, USA; 2 Department of Computer & Information Science, University of Pennsylvania, Philadelphia, USA; 3 Division of Pediatric General, Thoracic and Fetal Surgery, Children’s Hospital of Philadelphia, Philadelphia, USA; 4 Haptic Intelligence Department, Max Planck Institute for Intelligent Systems, Heisenbergstr. 3, 70569 Stuttgart, Germany

**Keywords:** Pediatric laparoscopic surgery, Intracorporeal suturing, Box trainer, Objective skill assessment, Motion analysis, Machine learning

## Abstract

**Background:**

Minimally invasive surgeons must acquire complex technical skills while minimizing patient risk, a challenge that is magnified in pediatric surgery. Trainees need realistic practice with frequent detailed feedback, but human grading is tedious and subjective. We aim to validate a novel motion-tracking system and algorithms that automatically evaluate trainee performance of a pediatric laparoscopic suturing task.

**Methods:**

Subjects (*n* = 32) ranging from medical students to fellows performed two trials of intracorporeal suturing in a custom pediatric laparoscopic box trainer after watching a video of ideal performance. The motions of the tools and endoscope were recorded over time using a magnetic sensing system, and both tool grip angles were recorded using handle-mounted flex sensors. An expert rated the 63 trial videos on five domains from the Objective Structured Assessment of Technical Skill (OSATS), yielding summed scores from 5 to 20. Motion data from each trial were processed to calculate 280 features. We used regularized least squares regression to identify the most predictive features from different subsets of the motion data and then built six regression tree models that predict summed OSATS score. Model accuracy was evaluated via leave-one-subject-out cross-validation.

**Results:**

The model that used all sensor data streams performed best, achieving 71% accuracy at predicting summed scores within 2 points, 89% accuracy within 4, and a correlation of 0.85 with human ratings. 59% of the rounded average OSATS score predictions were perfect, and 100% were within 1 point. This model employed 87 features, including none based on completion time, 77 from tool tip motion, 3 from tool tip visibility, and 7 from grip angle.

**Conclusions:**

Our novel hardware and software automatically rated previously unseen trials with summed OSATS scores that closely match human expert ratings. Such a system facilitates more feedback-intensive surgical training and may yield insights into the fundamental components of surgical skill.

**Electronic supplementary material:**

The online version of this article (doi:10.1007/s00464-017-5873-6) contains supplementary material, which is available to authorized users.

Minimally invasive surgery (MIS) has led to decreased postoperative pain, earlier hospital discharge, and decreased scarring compared to open surgery [1]. In several adult surgical procedures, such as appendectomy, MIS has become the recommended standard [2]. Despite the benefits of MIS methods, challenges remain in the development of training programs that effectively simulate operating conditions. While smaller incisions greatly benefit patients, minimally invasive methods introduce new ergonomic challenges for the training surgeon. Compared to open surgery, MIS requires planning for constrained space, the use of less intuitive movements through trocars, and the ability to interpret a three-dimensional surgical field from a two-dimensional video image [3]. Due to the unique nature of minimally invasive skills, an increased demand arose for synthetic models such as box trainers and virtual reality simulators that teach basic laparoscopic skills free of the pressures of operating on real patients [4].

Surgical simulators are widely regarded as safe and effective tools for practice and assessment during the early stages of a surgeon’s training. The use of box trainers and virtual reality simulators allows surgeons to improve their skills outside of the operating room and allows senior physicians to evaluate and correct the trainee’s approach without concern for a patient’s life. These artificial simulators have reproducible setups, allowing for longitudinal evaluation. Box trainers with realistic internal components such as artificial tissue are especially favored as training tools due to the fidelity of the physics of instrument handling and contact interactions and the realistically constrained workspace provided by the box. Previous studies [5–7] have validated the use of these box trainers to evaluate surgical skill, and others [8] have validated that skills acquired through simulation can be transferred to the operating room. Studies have also been done on virtual reality simulators, which were similarly able to predict surgical skill [9–11]. Although they play an important role in many training programs, virtual simulators are still not widely favored in comparison to box trainers because the physics of virtual reality are not robust enough to exactly mimic procedures [12]. The higher cost of virtual reality systems also affects their adoption.

Instrument motion tracking has been validated as a method of analysis for use in laparoscopic skills assessment [13–16], and the use of motion analysis to quantify surgical skill has further enhanced box trainers and allowed for quantification to accompany qualification of surgical skill [17]. The incorporation of motion analysis has allowed for more in-depth characterization of differences between novices and experts, although distinguishing skill on a finer scale that includes surgeons with intermediate capabilities continues to present a challenge [15]. As reviewed by Reiley et al. [17], the techniques of laparoscopic motion analysis have also been successfully adapted for evaluating trainee skill at robot-assisted MIS, where the surgeon operates the instruments through joystick-like hand controllers rather than through direct physical interaction. Moving beyond motion sensing, investigations have shown that statistical analysis of the vibrations of the robotic instruments and the forces applied to the inanimate task materials can be used to distinguish between novice and expert surgeons [18] and to rate different aspects of a trainee’s task performance on standardized 5-point scales [19].

Recently, MIS methods have been adapted for use on infants and small children [20–22]. While pediatric MIS offers many financial and medical advantages to the patient, training new surgeons in its techniques presents additional challenges compared to adult MIS. Specifically, the ergonomic constraints arising from a smaller working space, shorter and narrower tools, ports closer together, and an overall smaller margin of error make pediatric MIS particularly challenging [23]. For this reason, as MIS use has expanded in pediatric populations, training programs have sought to develop pediatric-specific training approaches and simulators. Small box trainers simulating the smaller pediatric body cavity have been developed and validated by Jimbo et al. [24] and Ieiri et al. [25]. Each group demonstrated the validity of their custom simulator using the Objective Structured Assessment of Technical Skills (OSATS) [26]. While this method of assessment has been fully established and validated for a range of simple and complex skills, implementation of OSATS to assess trainees requires a significant amount of time from a reviewer, and trainees rarely receive immediate feedback about their performance [27]. Thus, we and others are interested in the possibility of automating the assessment of surgical skills performed with real instruments on simulated tissue.

This article presents and validates a custom pediatric laparoscopic box trainer for use in a MIS training program at the Children’s Hospital of Philadelphia (CHOP). Specifically, we use a novel motion-tracking system, a corpus of recorded task performances, and video-reviewer-generated OSATS scores to create machine learning algorithms capable of automatically assigning scores to future performances of an intracorporeal laparoscopic suturing task. This system and its possible extensions may assist in the assessment of trainee skill while reducing the human cost of training and reviewing performances.

## Materials and methods

**Fig. 1:**
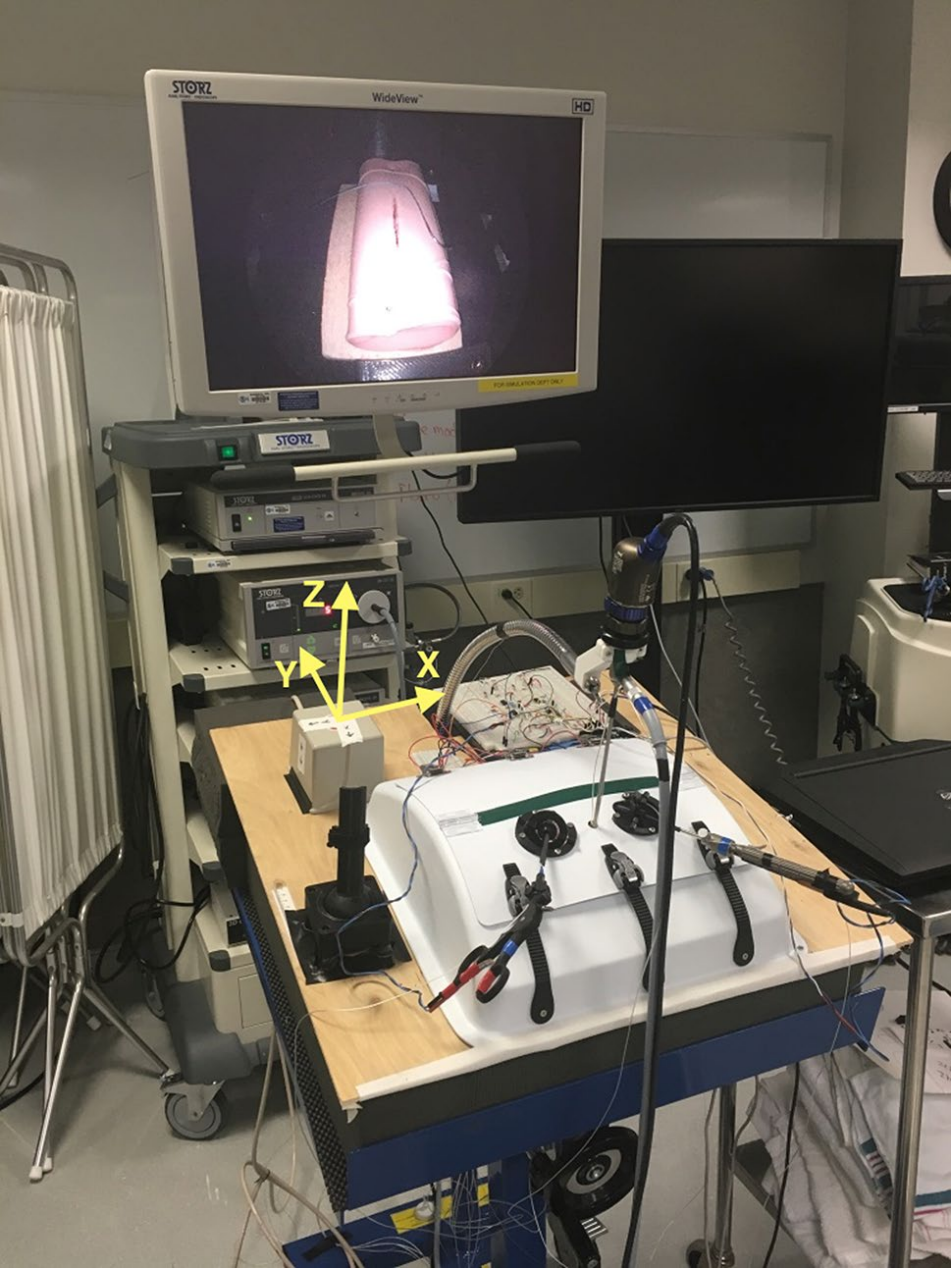
Full training setup

We created a custom apparatus for automatically evaluating trainee skill at a pediatric laparoscopic intracorporeal suturing task. Reaching this goal required us to build a suitable box trainer, instrument it with motion sensors, and write software for the user interface. The full setup is shown in Fig. 1. Evaluation of this training apparatus involved recruitment of trainee participants, recording a large number of task performances, rating each trial video on standardized scales, analyzing the recorded motion data, and building statistical models that can automatically rate new trials based only on recorded data.

### Custom laparoscopic box trainer

**Fig. 2:**
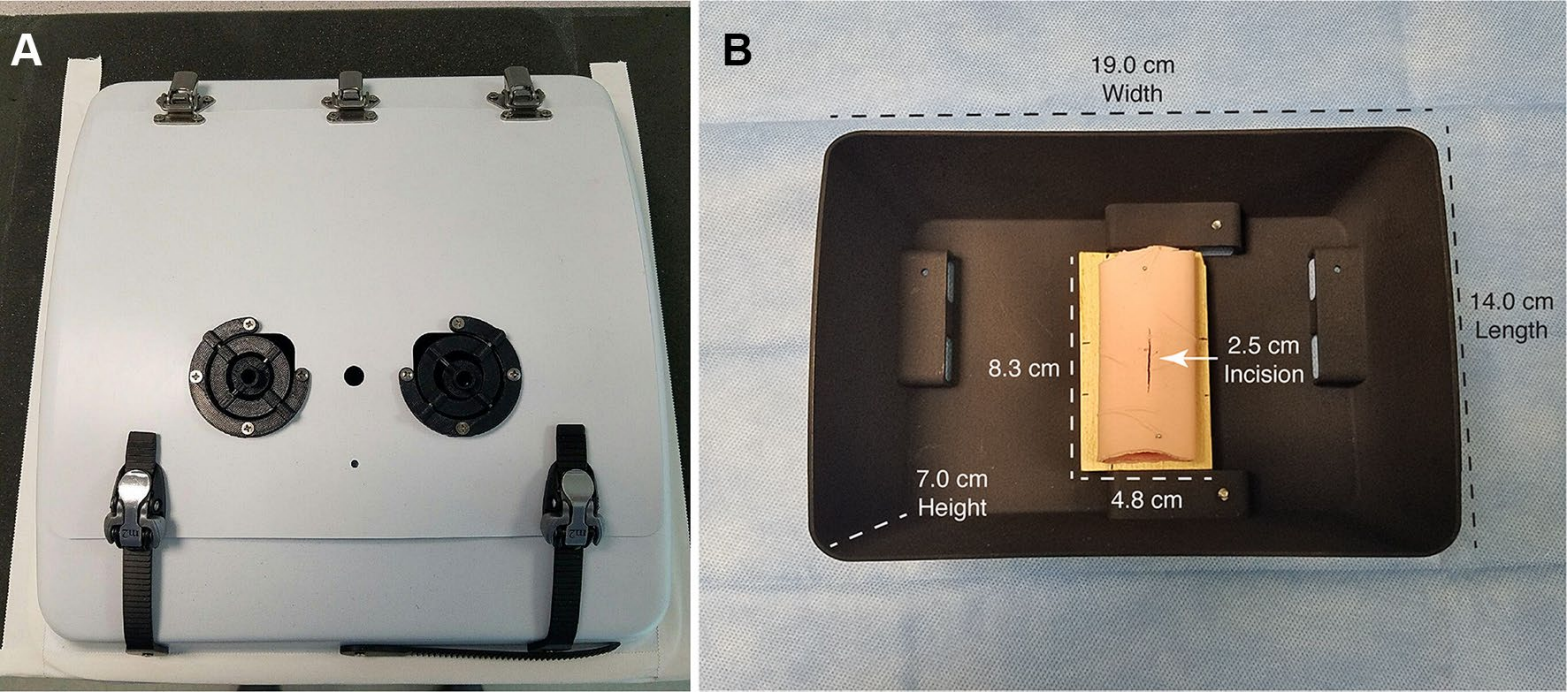
Custom laparoscopic box trainer: **A** exterior and **B** interior

Before creating an automatic skill assessment tool, we identified the need for a box trainer that more accurately represents the ergonomic challenges of pediatric surgery. Specifically, the box trainer needs to support the use of smaller instruments and provide the limited working space, visibility, and depth of a pediatric abdomen. As shown in Fig. 2A, a laparoscopic box trainer with exterior measuring 35.6 cm wide by 35.6 cm long by 7.0 cm tall was designed and manufactured with rigid sides and a compliant arched top surface. Custom trocars were created for compatibility with the pediatric box trainer out of 3D printed acrylonitrile butadiene styrene plastic and inserted into the top of the box trainer 4 cm from the center line of the workspace on each side. Each trocar gimbal measured 0.7 cm in diameter at the instrument insertion point. A 1-cm-diameter hole was placed at the midpoint between the two trocars to allow for insertion of an endoscope.

A length of simulated bowel (Tactility Simulated Bowel #2129, The Chamberlain Group) was cut to 7.6 cm and attached to an 8.3-cm-long by 4.8-cm-wide wood insert with two pins, one placed at each end of the artificial tissue. A 2.5-cm-long incision was cut into the center of the specimen. The simulated bowel was replaced before every trial for the sake of consistency. A removable 14-cm-long by 19-cm-wide by 7-cm-tall 3D printed insert was fabricated and used to secure the wood insert into the box trainer using hook-and-loop fasteners. The complete internal setup is shown in Fig. 2B.

**Fig. 3:**
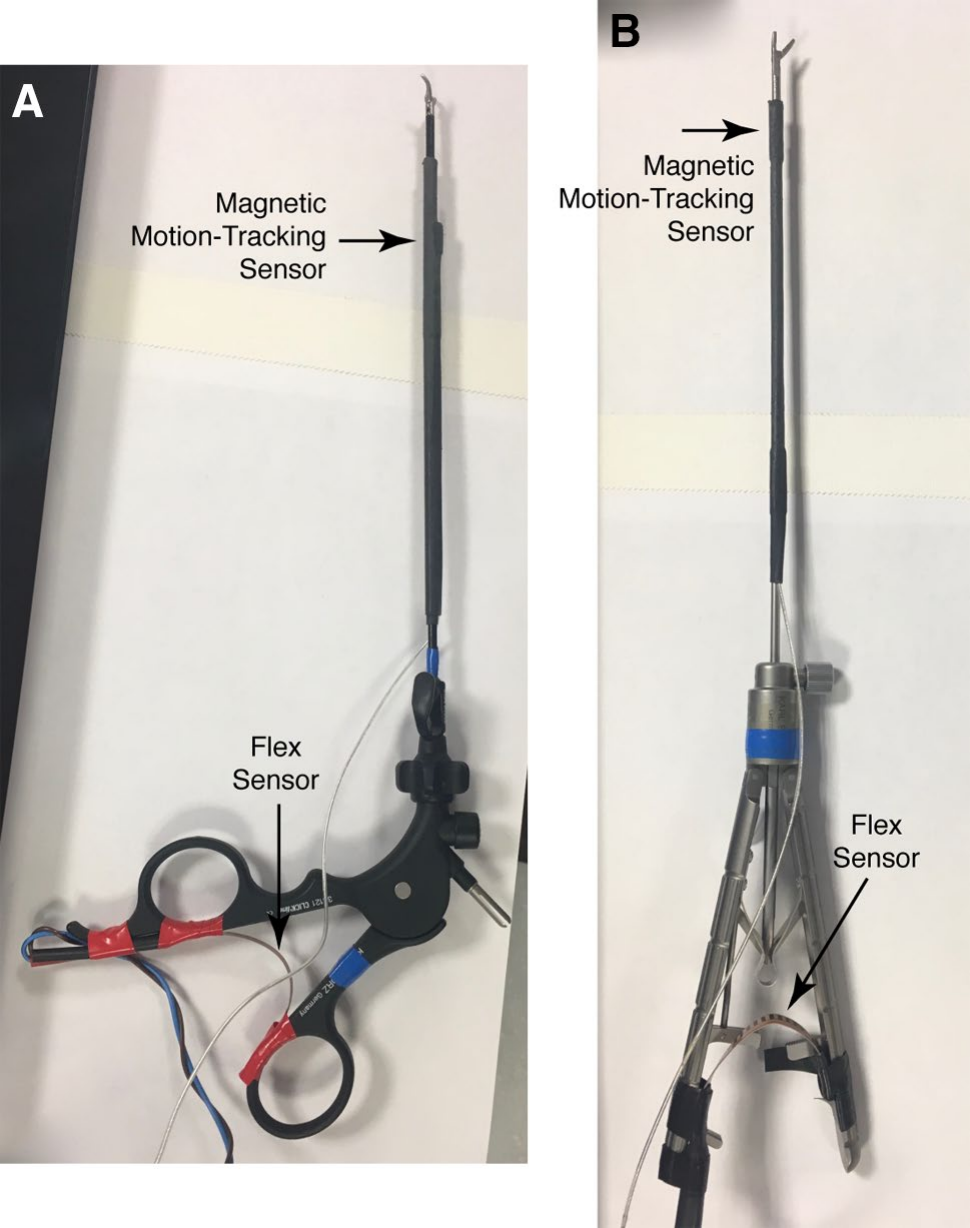
Each instrument shaft is equipped with a magnetic motion-tracking sensor, and each handle with a flex sensor. **A** Karl Storz CLICKline^®^ with Dissect/Grasp forceps tip (Maryland) and **B** Karl Storz Ultramicro Needle Holder (needle driver)

The surgical instruments and endoscope used in this study were the same tools employed for routine laparoscopic intracorporeal suturing at CHOP. As shown in Fig. 3, a 20-cm-long Karl Storz CLICKline^®^ instrument with 3.5 mm diameter and a 1-cm-long Dissect/Grasp forceps tip (also known as a Maryland) was placed in the left trocar, and a 20-cm-long Karl Storz Ultramicro Needle Holder (needle driver) with 3 mm diameter was placed in the right trocar. A 4-mm-diameter pediatric endoscope with 30° upward tilt (model 26009 BA, Karl Storz) was used for visualization of the surgical field. Recording of each trial was done by a Karl Storz HD Video Endoscopy System.

### Sensors

An Ascension trakSTAR 3D electromagnetic motion-tracking system with a mid-range transmitter was employed to track the motion of both tools and the endoscope; all measured motions were transformed to be relative to the coordinate frame shown in Fig. 1. A trakSTAR sensor (Model 130) was attached 2.5 cm from the tip of each laparoscopic tool aligned with the shaft, and an additional sensor was attached to the surface of the endoscope lens at a 30° angle from the shaft. The electromagnetic transmitter emits a magnetic field that each trakSTAR sensor measures to resolve all six degrees of freedom: three translational degrees of freedom (moving left/right, *T*_*X*_; moving forward/backward, *T*_*Y*_; and moving up/down, *T*_*Z*_) as well as three rotational degrees of freedom (tilting up/down, *R*_*X*_; rotating around the tool axis, *R*_*Y*_; and bending left/right, *R*_*Z*_). The position and orientation of all three sensors are measured with the resolutions of 1.4 mm and 0.5° at a rate of approximately 20 Hz.

Mounting the magnetic motion sensors directly on the metallic surface of each instrument’s shaft resulted in poor signal quality; adding a layer of rubber heat-shrink tubing beneath the sensor improved the signal quality to the recommended level. A thin layer of electrical tape and an outer layer of heat shrink were used to fasten the magnetic sensor and wiring rigidly to each tool. Furthermore, the table on which the box trainer rests is metallic, which was found to distort the motion tracking somewhat. However, we wanted to use this table rather than a non-metallic table of fixed height to allow each participant to adjust the setup for proper ergonomics. Thus, we placed a 7.6-cm-thick block of foam between the adjustable metal table and covered it with a thin piece of wood to which the box trainer was then mounted.

In addition to tool tip motion, we wanted to record each instrument’s final degree of freedom: opening and closing of the handle, which controls grasping motion and forces. It is important that this sensor does not impede normal tool use and movement during the task. For this application, a 5.6-cm-long flex sensor (Spectra Symbol SEN-10264) was fastened to the inside of the handle of each tool using electrical tape, as shown in Fig. 3. The resistance of the flex sensor increases as it bends, enabling us to measure grip angle. The two corresponding analog voltages were sampled by an Arduino Uno (ATmega328P) microcontroller and communicated to the computer via universal serial bus (USB) at 20 Hz.

### Calibration

**Fig. 4:**
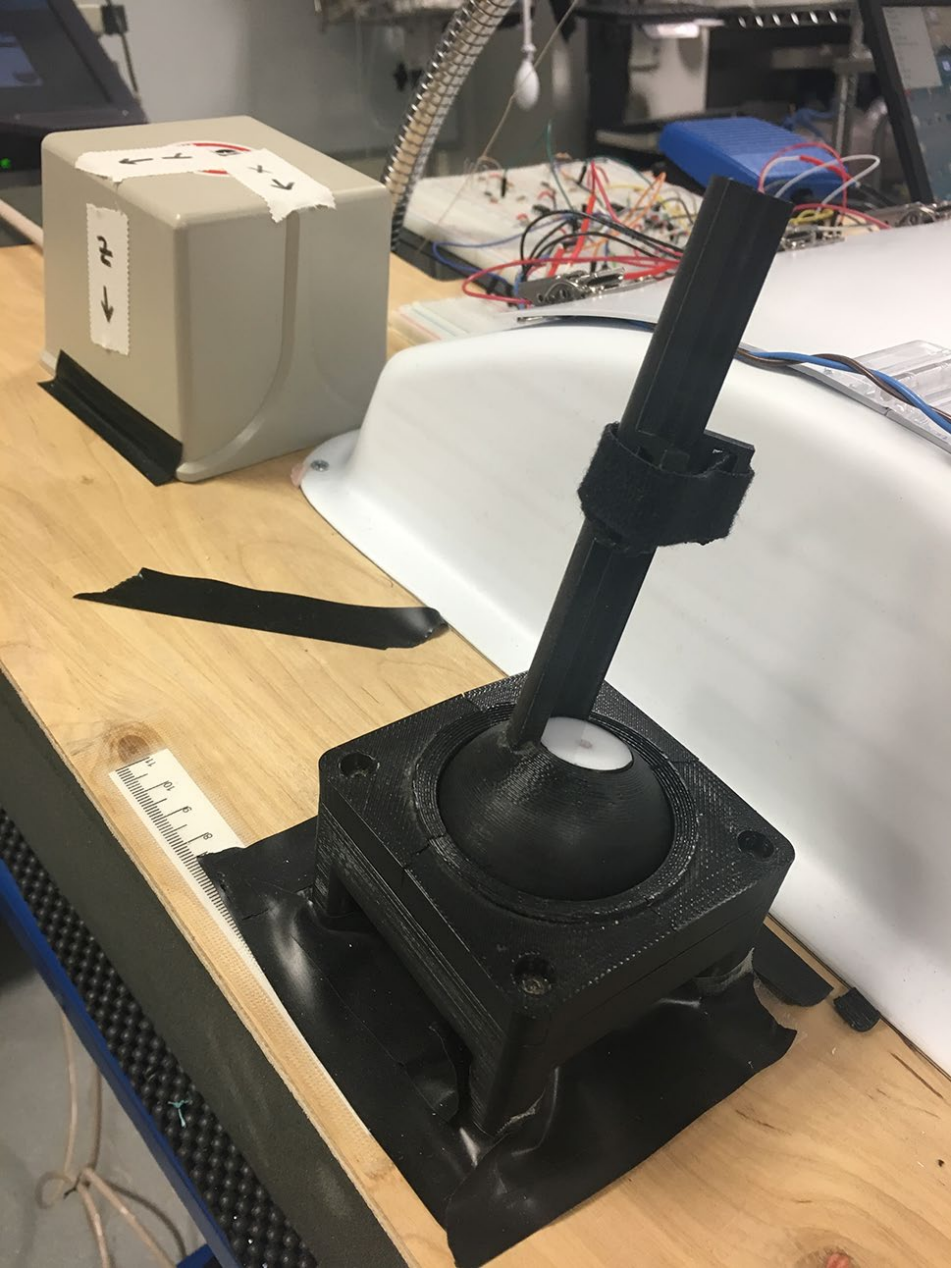
The device used to calibrate the placement of the trakSTAR sensors on the instruments. During use, the tool tip is inserted into the white mount, and the tool shaft is strapped to the vertical portion, which can rotate to all angles via a spherical joint centered around the tool tip

The tool-mounted trakSTAR sensors were calibrated before the study to identify the location of the instrument tip relative to the position and orientation of the sensor on the instrument shaft. Calibration was checked after every five participants or so to verify that the heat-shrink tubing kept each sensor rigidly attached. A previously developed device [28] was adapted to perform calibration, as shown in Fig. 4. The calibration rig was rigidly attached to the wood table surface directly to the left of the box trainer and within the range of the trakSTAR transmitter. Circular disks were placed in the rig to steady each tool during the calibration procedure, and the tool was fastened into the rig using a strap. The rig keeps the tool tip stationary as the user rotates the handle to many different positions and orientations. We recorded 30 s of such motion, and a hemisphere was then fit to the recorded positions of the sensor. A distance vector from any point on the hemisphere to the center represents the displacement from the sensor to the tip of the instrument, and the recorded orientation of each sensor pose on the hemisphere was used to calculate the orientation of the sensor relative to the instrument shaft. The results of calibration matched up well with direct measurements.

Each flex sensor was calibrated prior to every subject’s first trial to account for any slight changes in its measurements. For the Maryland, calibration involved holding the tool open for 5 s followed by holding it fully closed for 5 s. Calibration of the needle driver involved holding the tool open, closed without engaging the ratchet, and fully ratcheted closed for 5 s each. The voltages measured during these calibration activities were used to convert flex sensor voltages to grip angles for each trial.

The trakSTAR sensor on the endoscope was calibrated before the study began to determine the camera’s field of view. The basic premise for this calibration was the least squares fitting of a cone to the field of view. A circle of known radius was printed on a sheet of paper and fastened to a piece of cardboard. The circle was then held at two different distances away from the camera parallel to the lens, and a separate trakSTAR sensor was used to trace the circle at each of these distances. The camera’s viewing axis was estimated by holding the free sensor in the center of the field of view. The positions of the two traced circles and the estimated axis were used calculate the size, position, and orientation of the viewable cone relative to the sensor on the endoscope. The resulting geometry corresponded well with direct measurements.

### User interface

**Fig. 5:**
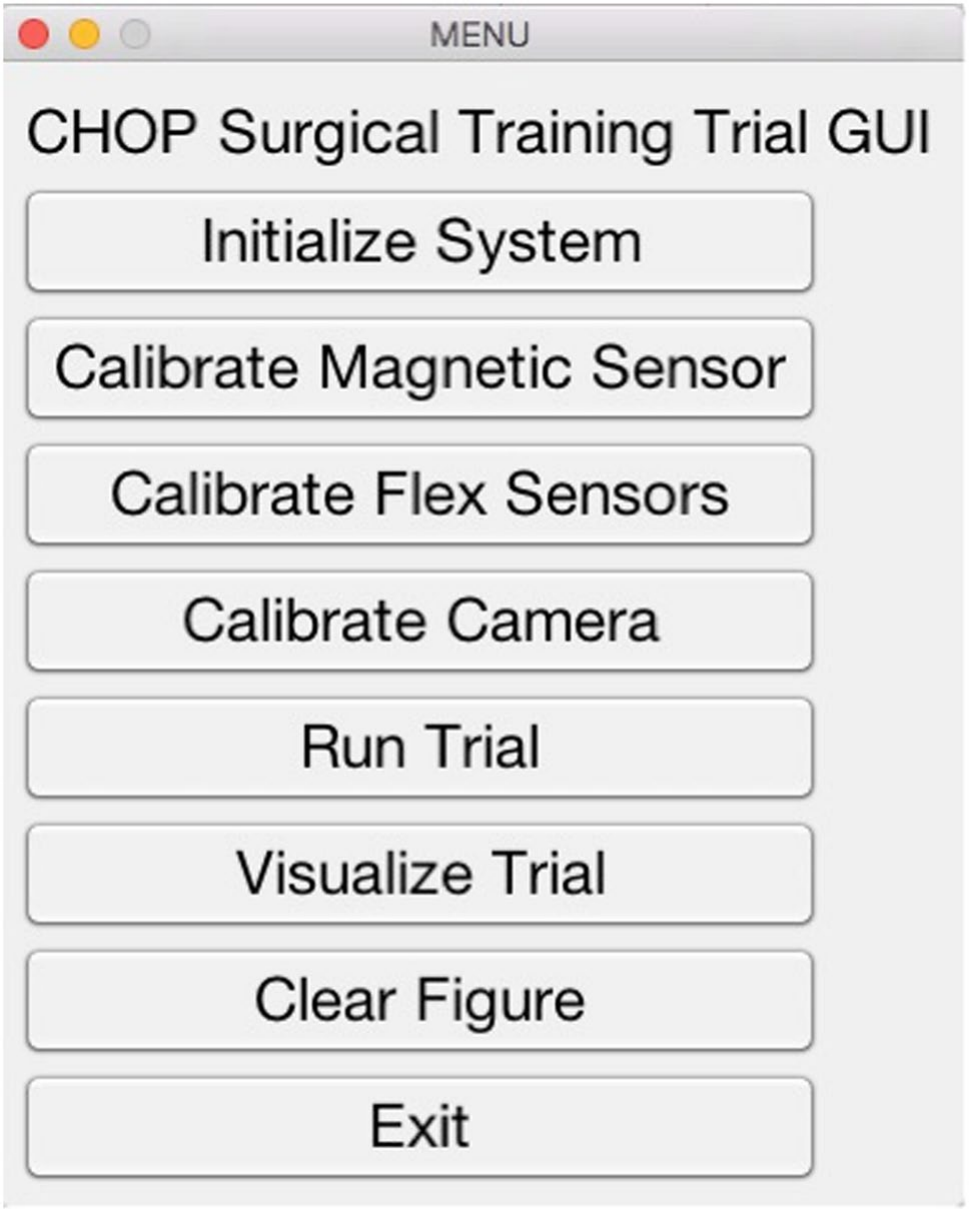
Graphical user interface

All data were recorded, timestamped, and saved using MATLAB version 2016a (The MathWorks). The software we created allows the user to calibrate the sensors, enter information about the participant, and set up data recording. A screenshot of the interface is shown in Fig. 5. A foot pedal on the ground and red light-emitting diodes (LEDs) in the box trainer were used for controlling data recording.

### Recruitment of subjects

Study procedures were reviewed by the CHOP Institutional Review Board (IRB). The IRB determined the study to be exempt from further review due to its educational nature, the fact that no patients were enrolled or involved, and the lack of compensation for participants. Subjects were recruited from the University of Pennsylvania Hospital System and the Children’s Hospital of Philadelphia. Recruitment methods included word-of-mouth, e-mail, and verbal announcement during educational sessions in the Pediatric Endoscopic Surgical Training and Advancement Laboratory (PEDESTAL). Medical students, surgical residents, and surgical fellows with experience in laparoscopic surgery were asked to participate; a total of 32 subjects were enrolled in the study. Table 1 shows the demographics of the recruited pool of participants.

**Table 1:** Participant demographics. One subject completed only one trial, and the rest did two. Several participants had experience with more than one task requiring bimanual dexterity

Number of participants	32
Number of trials recorded	63
Age in years	
1st quartile: 24–27	9
2nd quartile: 28–29	8
3rd quartile: 30–31	7
4th quartile: 32–36	8
Gender	
Female	17
Male	15
Level of training	
Medical student	6
Resident	21
Fellow	5
Number of laparoscopic procedures performed in the month prior to trial	
0	7
> 0 and ≤ 10	8
> 10 and ≤ 50	12
> 50 and ≤ 100	3
> 100	2
Surgical glove size	
5.5	2
6	5
6.5	9
7	11
7.5	4
8	1
Handedness	
Left handed	0
Right handed	31
Ambidextrous	1
Experience with tasks requiring manual dexterity	
Sports	3
Musical instruments	15
Video games	6
None	11

### Task and procedure

**Fig. 6:**
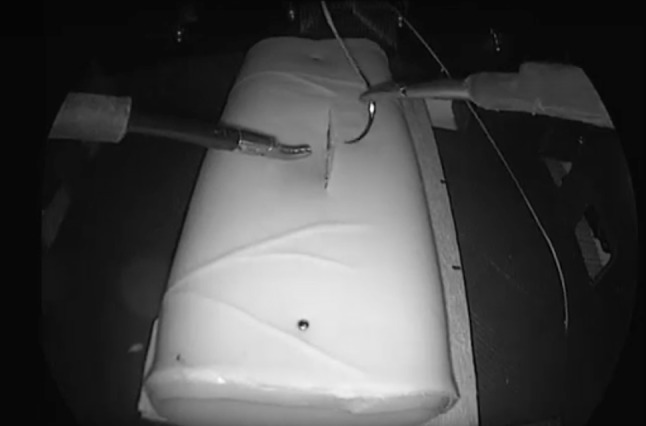
Frame from a sample video of the task, showing the visibility of the tools in the foreground, the typical camera view quality, and LEDs illuminated in the background, which indicate that the trial data are being recorded

Participants first watched an un-narrated but visually annotated video of ideal task performance by an expert; a narrated copy of this video is included with this article. The subject was then asked to use the custom box trainer to tie one double-throw knot followed by two single-throw knots with an 8-cm-long 2.0 Ethibond suture on an RB-1 needle to close the incision in the simulated bowel. Clarification was allowed prior to data recording, but no instruction or clarification was allowed once the first trial was initiated. A member of the study team placed the suture directly on top of the pre-cut artificial tissue and instructed the subject to grasp the needle prior to starting the trial. Video recording began when the subject first held the tools. The subject was instructed to tap the start pedal when he or she was ready to begin the task, which triggered the computer to begin recording data from all sensors. Illumination of LEDs inside the box trainer indicated that data collection had begun. Participants were not observed during the task in order to prevent later rating bias. Data acquisition ended and the LEDs turned off when the subject tapped the pedal a second time. Trials were capped at 10 min, and data acquisition was automatically terminated at that time for subjects who had not yet completed the task. All but one participant attempted the task twice, with a brief break between trials. Figure 6 shows a still frame from a sample performance video. In total, 63 trials were recorded.

### Video review and OSATS ratings

The skill assessment scores used in this study were on a scale from 1 to 5 in each of five categories taken from the OSATS inventory [29]. The chosen categories included Respect for Tissue (how appropriately the trainee handled tissue), Time and Motion (economy and efficiency of movement), Instrument Handling (fluidity of instrument movements), Flow of Operation (fluidity with which task was completed), and Knowledge of Specific Procedure (measure of whether all steps were completed). The Use of Assistants and Knowledge of Instruments OSATS domains were excluded from analysis because they are not relevant for an unassisted task that uses the same instruments throughout.

All recorded videos were de-identified and randomized prior to review. Only the segment of the video where the LEDs were illuminated was viewed and rated. A surgical research fellow was trained by an attending surgeon and then assigned five scores for each trial, yielding a total of 315 ratings. These scores are the ground-truth labels for our machine learning pipeline, which seeks to rate surgical skill based on only the recorded motion data. The five categorical ratings were turned into two scores for each trial, one being a sum of the scores in the five categories and the other being a rounded average of the scores in the five categories.

### Analysis of motion parameters

We sought to reduce the time-varying motion data recorded in each trial to a smaller set of meaningful values for further analysis. Raw motion data were filtered using a fourth-order two-way low-pass Butterworth filter with a cutoff frequency of 6 Hz to eliminate noise. The data were then re-sampled to exactly 20 Hz to account for any small differences in sampling frequency between trials. 280 motion analysis features (MAFs) were designed and then computed for each trial. Motion analysis was split into several categories based on the sensor from which the MAF was derived. These categories included trial time (*T*), linear and angular tool tip motion (*M*), tool tip visibility (*V*), and grip angle (*G*).

Time MAFs included the duration of the trial as well as the square, square root, inverse, and squared inverse of this value. Linear and angular tip motion features were derived directly from the position and orientation of each instrument. Features in these two categories were further divided into the subcategories of path length, velocity, and acceleration overall and in each Cartesian direction. Velocity and acceleration categories were further divided into instantaneous and average subcategories. For each of the instantaneous subcategories, features included maximum and minimum instantaneous values, range of instantaneous values, and average values. Tool tip visibility MAFs included the ratio of time that each instrument spent within the camera view versus outside of the camera view. Grip angle features include the number of instantaneous peaks, instantaneous velocity of grip flexion, and instantaneous acceleration of grip flexion for each tool. For completeness, the calculated MAFs are summarized in Table 2.

**Table 2:** Motion analysis features (MAFs)

Sensor type	Motion analysis features
Time (calculation time: 0.03 s)	Total trial time
	Inverse of trial time
	Inverse square of trial time
	Square of trial time
	Square root of trial time
Tool tip visibility (calculation time: 0.33 s)	Time inside camera view Time outside camera view Time visible/(time visible + time not visible)
Grip (calculation time: 0.57 s)	Velocity (Peaks ≥ 5°/s)/trial time (Peaks ≥ 10°/s)/trial time (Peaks ≥ 20°/s)/trial time Time *v* = 0 Time *v* > 0 Time *v* = 0/(time *v* = 0 + time *v* > 0) Acceleration (Peaks ≥ 100°/s^2^)/trial time Time *a* = 0 Time *a* > 0 Time *a* = 0/(time *a* = 0 + time *a* > 0)
Tip motion (calculation time: 12.43 s)	Linear path X, Y, and Z path lengths (for each tool) Total path length (for each tool) Path range (for each tool) Ratios of values from each tool Angular paths Roll, elevation, and azimuth angular path lengths (for each tool) Total angular path length (for each tool) Angular path range (for each tool) Ratios of values from each tool Average linear speed *Vx*_*a*_, *Vy*_*a*_, and *Vz*_*a*_ (for each tool) Total average speed (for each tool) Ratios of values from each tool Instantaneous linear velocity *Vx*_*i*_, *Vy*_*i*_, and *Vz*_*i*_ (for each tool) Max, min, and std. dev. of each value (for each tool) Ratios of values from each tool (Peaks ≥ 2 cm/s)/trial time (Peaks ≥ 5 cm/s)/trial time (Peaks ≥ 10 cm/s)/trial time (Peaks ≥ 100 cm/s)/trial time Average angular speed *ωx*_*a*_, *ωy*_*a*_, and *ωz*_*a*_ (for each tool) Total average speed (for each tool) Ratios of values from each tool Instantaneous angular velocity *ωx*_*i*_, *ωy*_*i*_, and *ωz*_*i*_ (for each tool) Max, min, and std. dev. of each value (for each tool) Ratios of values from each tool (Peaks ≥ 1°/s)/trial time (Peaks ≥ 10°/s)/trial time (Peaks ≥ 20°/s)/trial time (Peaks ≥ 200°/s)/trial time Average linear acceleration *ax*_*a*_, *ay*_*a*_, and *az*_*a*_ (for each tool) Total average speed (for each tool) Ratios of values from each tool Ratios of values from each tool Instantaneous linear acceleration *ax*_*i*_, *ay*_*i*_, and *az*_*i*_ (for each tool) Max, min, and std. dev. of each value (for each tool) Ratios of values from each tool (Peaks ≥ 10 cm/s^2^)/trial time (Peaks ≥ 100 cm/s^2^)/trial time (Peaks ≥ 1000 cm/s^2^)/trial time (Peaks ≥ 10,000 cm/s^2^)/trial time Average angular acceleration *αx*_*a*_, *αy*_*a*_, and *αz*_*a*_ (for each tool) Total average speed (for each tool) Ratios of values from each tool Instantaneous angular acceleration *αx*_*i*_, *αy*_*i*_, and *αz*_*i*_ (for each tool) Max, min, and std. dev. of each value (for each tool) Ratios of values from each tool (Peaks ≥ 50°/s^2^)/trial time (Peaks ≥ 100°/s^2^)/trial time (Peaks ≥ 1000°/s^2^)/trial time (Peaks ≥ 10,000°/s^2^)/trial time

Calculation of this full set of 280 MAFs took an average of 13.29 s on a 2011 MacBook Pro with a 2.3 GHz Intel Core i5 processor and 4 GB of RAM. Calculation of the 247 tip motion MAFs was the most time-intensive, taking 12.43 s. In comparison, calculating the five time MAFs took 0.03 s, calculation of the 8 tool tip visibility MAFs took 0.33 s, and calculation of the 20 grip angle MAFs took 0.57 s.

### Statistical analysis

Six models were created to predict summed OSATS score based on the following different subsets of MAFs:

- Model T: time MAFs
- Model TG: time and grip MAFs
- Model TM: time and tip motion MAFs
- Model TMV: time, tip motion, and tool visibility MAFs
- Model TMG: time, tip motion, and grip MAFs
- Model TMVG: time, tip motion, visibility, and grip MAFs

Prior to analysis, the feature space of the training set was standardized by subtracting the mean and dividing by the standard deviation of each MAF. Each model was then trained to predict the sum of the five 5-point scores given by the human rater based only on these standardized motion features. As 280 features is a high number, the least absolute shrinkage and selection operator (LASSO) elastic net technique [30] was used to mathematically identify the most relevant features in the training set for each of the six predictive models. The resulting smaller set of features was used to train a regression tree model for each OSATS category. To create models that can generalize more effectively to data from new participants, we used leave-one-subject-out cross-validation. Each model is trained and optimized on the data from all but one subject, and its performance is then evaluated on the reserved trials, a process that repeats across all subjects to yield a performance average based on all collected data. We examine both summed score and rounded average score.

For each way of predicting a trainee’s score, we report six accuracy metrics; each metric varies from 0 to 1, with 1 being best. For summed score, we report the proportion of test trials for which the model predicted a score within 2 points of the reviewer-generated score; for example, a trial that received a summed rating of 15 would need to be given a 13, 14, 15, 16, or 17 to be counted as correct according to this measure. As a similar but more lenient metric, we next report the proportion of trials for which the predicted summed score fell within 4 points of the actual score. Third, we calculated the Pearson product-moment correlation coefficient between the actual and predicted summed scores. For rounded average scores, we report the proportion of test trials that were rated with exact accuracy (error of 0 points), as well as the proportion that fell within 1 point on the 4-point scale. Finally, we present the correlation between actual and predicted rounded average scores.

Two simple control models were also created for comparison with our regression-based methods. The first model guessed randomly, which is expected to perform poorly, and the second predicted the median observed score on every trial, which is expected to perform only slightly better. Each of the six metrics described above was also calculated for these models, except that by definition one cannot calculate a correlation for the median model because its output does not vary.

## Results

### OSATS ratings

**Fig. 7:**
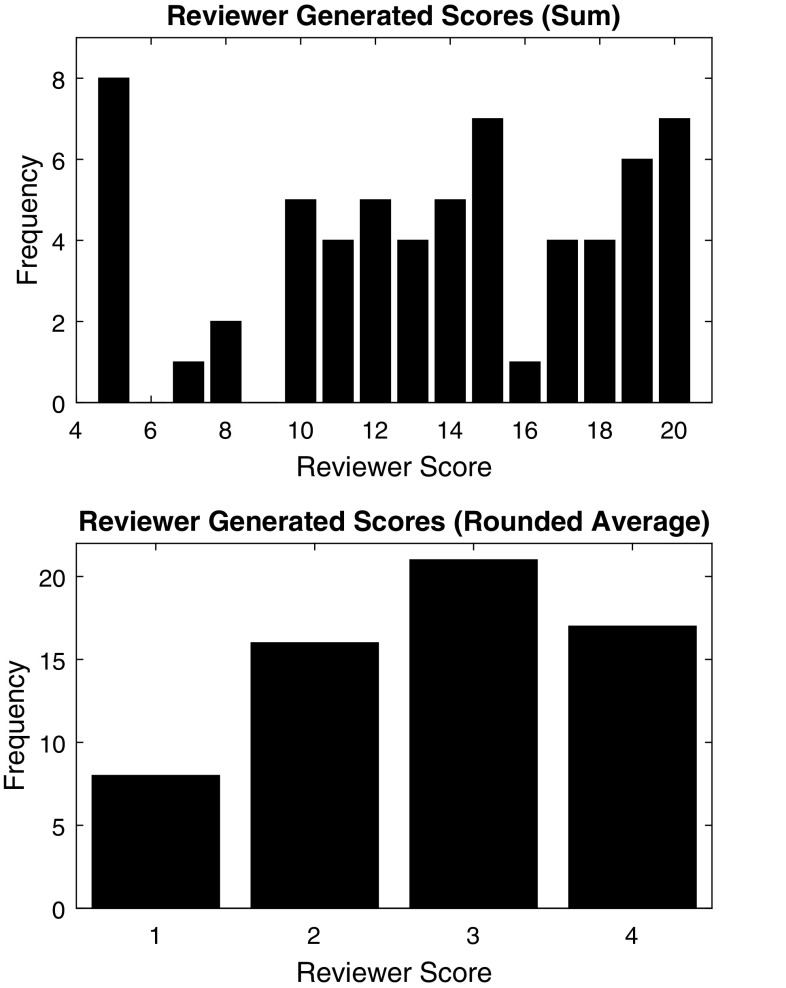
OSATS scores generated by the blinded expert video reviewer for all 63 trials. Top: summed scores, bottom: rounded average scores

The blinded expert reviewer rated each recorded trial video on five OSATS domains using the standard 1–5 scale; however, the rater identified no trials that merited a 5 in any category, presumably because no attending surgeons took part in this study. The five resulting ratings from 1 to 4 were then summed to generate a total skill score that ranges from 5 to 20. The distribution of these summed scores for all 65 trials is shown in the top half of Fig. 7. The mode of the summed scores was 5 out of 20 (*n* = 8), and the median was 14. We also calculated each trial’s average integer OSATS score by dividing the summed score by 5 and rounding; the resulting score distribution is shown in the bottom half of Fig. 7. The mode of the rounded average scores was 3 (*n* = 21), and the median was also 3. To help validate these ratings, we also examined their distribution for each training level, as shown in Table 3. The mean and standard deviation rounded average OSATS scores were 1.33 ± 0.49 for medical students, 2.93 ± 0.49 for residents, and 3.60 ± 0.52 for fellows.

**Table 3:** Distribution of rounded average OSATS scores for the three groups of trainees who participated in the study

Level of training	1	2	3	4	Mean ± std. dev.
Medical students (12 trials)	8	4	0	0	1.33 ± 0.46
Residents (41 trials)	1	12	17	11	2.93 ± 0.49
Fellows (10 trials)	0	0	4	6	3.60 ± 0.52

**Fig. 8:**
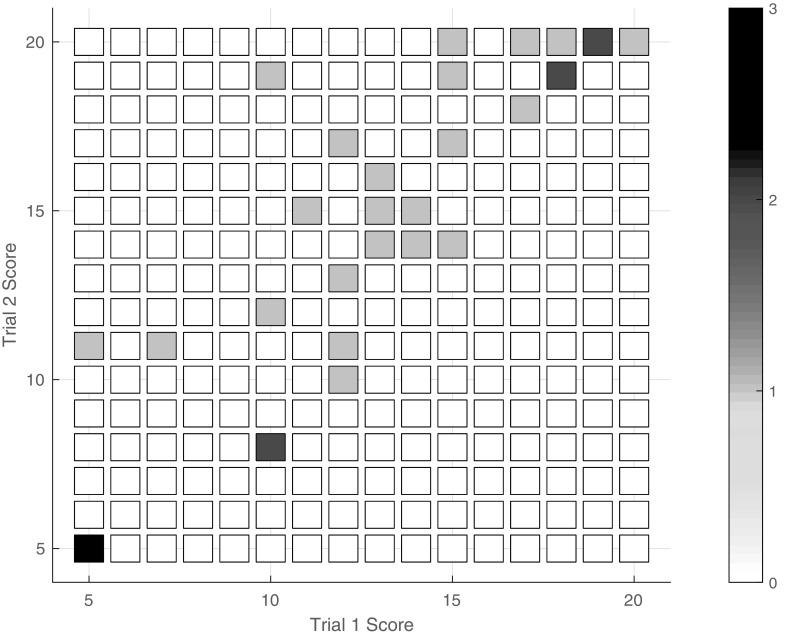
Relationship between first trial summed score and second summed trial score for all subjects who completed two trials (*n* = 31)

One participant completed only a single repetition of the simulated pediatric suturing task, earning a summed score of 5, which corresponds to an average OSATS score of 1. The rest of the subjects (*n* = 31) completed two trials of the task, so we can examine the trends in their individual scores. Figure 8 shows the reviewer-generated score of each subject’s first trial plotted against the score assigned to the same subject’s second trial. The Pearson product-moment correlation coefficient between these two metrics is *R* = 0.89, indicating a high correlation. Five participants (16.1%) showed a slight decrease in performance on the second trial, four (12.9%) had no change in performance, and 22 (71.0%) improved their summed score. Individuals who first scored at either the bottom or the top of the range tended to earn a similar score on the second trial. In contrast, subjects who first earned between 10 and 15 out of 20 showed the most improvement.

### Automatic scoring performance

A combination of regularized least squares regression (LASSO) and regression trees was used to create models that can predict the summed OSATS score of a previously unseen trial using only the data recorded from our instrumented box trainer. We label the four employed sensors as the trial completion timer (*T*), the two flex sensors that measure the grip angle of the instrument handles (*G*), the magnetic motion tracking of the instrument tips (*M*), and the visibility of the instrument tips to the camera (*V*). We explored the relative contributions of these different sensors by creating six regression models using different combinations of the 280 total features, yielding T, TG, TM, TMV, TMG, and TMVG models.

As described in the previous section, each regression tree model was trained 32 separate times, always leaving out the data from one subject. The six models were all optimized to achieve the best possible performance on the training set data through cross-validation. We also report results from the random model, which guesses a summed score from 5 to 20 with uniform probability, and the median model, which always guesses the median score (14 out of 20 for summed and 3 out of 5 for average). Table 4 shows the average performance of these eight model types on the training data. All of the regression models achieved good results on the training data, even the simplistic Model T. But to test our approach’s ability to rate data from new trials, each of the resulting models was then used to predict scores for its respective omitted trials (the testing set), and the results were averaged across subjects. Table 5 reports the results achieved by the eight models for predicting summed as well as rounded average OSATS scores on the testing data.

**Table 4:** Averaged automatic scoring performance for the eight models on the training data

Model	Summed scores			Rounded average scores		
	± 2 Accuracy	± 4 Accuracy	Correlation	± 0 Accuracy	± 1 Accuracy	Correlation
Random	0.28	0.47	< 0.01	0.27	0.69	0.01
Median	0.35	0.62	NaN	0.33	0.86	NaN
T	0.97	1.00	0.91	0.88	1.00	0.86
TG	0.95	0.99	0.95	0.86	1.00	0.92
TM	0.98	1.00	0.96	0.88	1.00	0.93
TMV	0.94	0.99	0.98	0.85	1.00	0.94
TMG	0.89	0.99	0.97	0.84	1.00	0.93
TMVG	0.77	0.98	0.98	0.73	1.00	0.94

The abbreviations indicate which features are included in each model: *T* time, *G* grip angle, *M* tip motion, and *V* tool visibility. NaN signifies “not a number” and occurs because correlation with a constant rating is undefined

**Table 5:** Averaged automatic scoring performance for the eight models on testing data from participants whose data were not used during training

Model	Summed scores			Rounded average scores		
	± 2 Accuracy	± 4 Accuracy	Correlation	± 0 Accuracy	± 1 Accuracy	Correlation
Random	0.24	0.48	< 0.01	0.25	0.68	0.03
Median	0.35	0.62	NaN	0.33	0.86	NaN
T	0.52	0.78	0.69	0.44	0.94	0.69
TG	0.52	0.68	0.68	0.54	0.95	0.68
TM	0.46	0.60	0.42	0.38	0.81	0.42
TMV	0.59	0.83	0.78	0.51	0.97	0.78
TMG	0.54	0.70	0.59	0.49	0.89	0.60
TMVG	0.71	0.89	0.85	0.59	1.00	0.85

The abbreviations indicate which features are included in each model: *T* time, *G* grip angle, *M* tip motion, and *V* tool visibility. NaN signifies “not a number” and occurs because correlation with a constant rating is undefined

As expected, the random model performed worst on all metrics for both training and testing. The somewhat more intelligent median model performed consistently worse than all six of the machine learning models, with only two exceptions: the testing accuracy of Model TM was worse than that of the median model on both of the more lenient accuracy metrics (predicting summed score within 4 points and rounded average score within 1 point). Of the six regression models, the one that tested worst was Model TM, which estimates the score using only the task completion time and the tool tip motion features.

At the other end of the spectrum, the model with the best performance on all six testing accuracy metrics was Model TMVG, which uses features from all four sensor types to estimate the score for a given trial. It predicted a summed score that was within 4 points of the reviewer’s summed score 89% of the time, and its rounded average scores were always within 1 point of the true label. The correlation between the reviewer’s scores and those of this predictive model was 0.85 for both score types.

Despite the relatively poor performance of the model that considered only time and tip motion data, the second best performing model behind TMVG was Model TMV, which added just eight tool visibility features to the set available to Model TM. This model estimated a summed score within 4 points of the reviewer-generated score 83% of the time, and its rounded average score was within 1 point of the reviewer’s score 97% of the time. After Model TMV, the next best performing model overall was Model TG, which used only time and grasp angle features. This model especially benefited from the class boundaries, predicting rounded average score within 1 point 95% of the time.

**Fig. 9:**
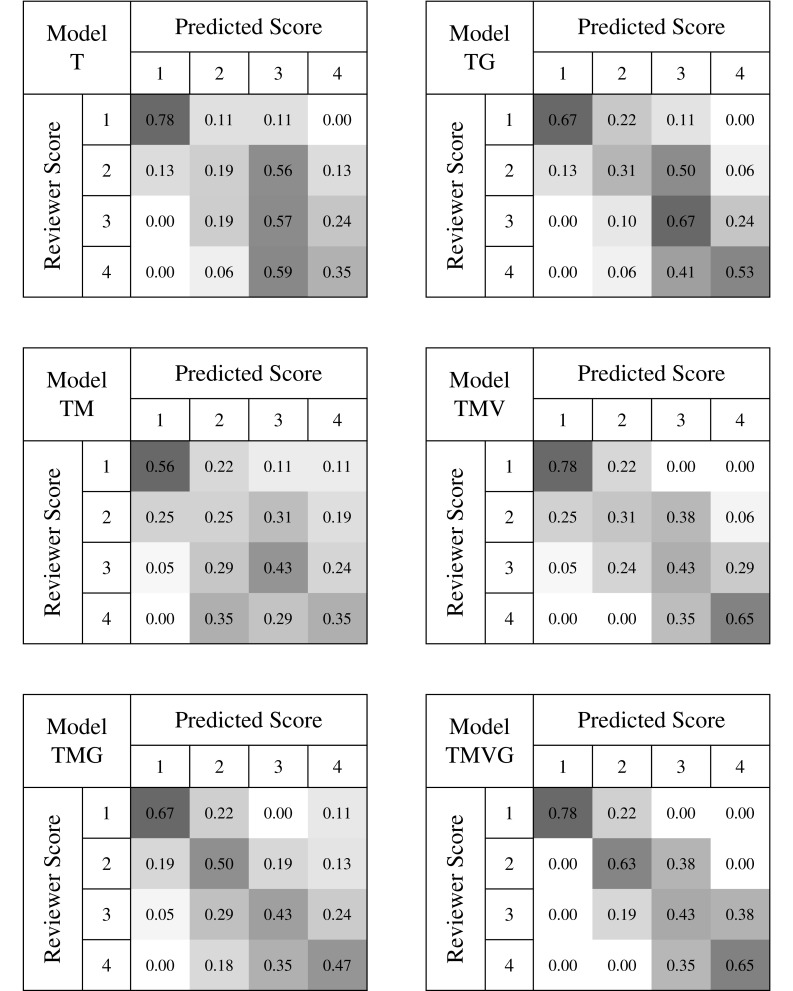
Confusion matrices for the six models generated from motion data. The abbreviations indicate which features are included in each model: *T* time, *G* grip angle, *M* tip motion, and *V* tool visibility. Each cell represents the number of testing trials predicted to have the score in the column divided by the total number of trials given the score in that row by the reviewer. The color intensity of each cell is proportional to the value of the cell on a scale from 0 to 1

The performance of each model was also visually evaluated through the creation of four-by-four confusion matrices, as shown in Fig. 9. These plots illustrate how the automatically generated rounded average ratings compare to the reviewer-generated scores in each category for the testing sets; perfect performance would entail that all trials fall along the main diagonal. These plots show that Models T and TM have the widest distribution of scores, while the high-performing Model TMVG shows the best score distribution, with no errors greater than 2 points. All of the sensor-and-regression-based models seem moderately successful at identifying trials that deserve a rating of 1, but distinguishing between 2’s, 3’s, and 4’s appears to be more challenging.

### Features selected

While up to now we have been focusing on the accuracy achieved when using different types of sensor data to rate surgical skill, the regression models also contain important information about the aspects of the data that were most important for the rating. Namely, each model selected a different subset of features for the final regression tree analysis. Table 6 provides the number of each type of feature employed in each model, as well as the total number of available features in each category. Because of the low dimensionality of their feature spaces, Models T and TG selected all and nearly all of their available features, respectively. In contrast, Models TM and TMV selected the highest numbers of features (over 100). While the addition of visibility features increased the number of features selected for Model TMV, the addition of grasp angle features slightly decreased the number of features selected in Model TMG. Of the models that involve tool tip motion, Model TMVG selected the fewest features. With the exception of one feature in Model TMV, Models TM, TMV, TMG, and TMVG included none of the features based on task completion time.

**Table 6:** Number of features included in each model out of the total number of features available in that category

Model	Overall	Time	Motion	Visibility	Grasp
T	5/5	5/5	-	-	-
TG	24/25	5/5	-	-	19/20
TM	101/252	0/5	101/247	-	-
TMV	107/260	1/5	100/247	6/8	-
TMG	95/272	0/5	89/247	-	6/20
TMVG	97/280	0/5	77/247	3/8	7/20

The abbreviations indicate which features are included in each model: *T* time, *G* grip angle, *M* tip motion, and *V* tool visibility. A hyphen appears when a given model does not employ sensor data of the specified type

Of the eight possible visibility features, the three that were selected by both TMV and TMVG included the time that the needle driver was within the camera frame, the ratio of the time the needle driver was in frame over the total time in the trial, and the ratio of time that the needle driver was out of frame over the total time in the trial. While these same features appear for the Maryland grasper in Model TMV, they are not used within Model TMVG. Looking further into the selected features, we found that all models that included grip angle data selected features that consider the ratio between the amounts of time each handle was engaged versus held stationary. Additionally, these models tended to select features that analyzed the number of peaks in the grip angle velocities.

The tip motion features (M) are divided into ten subcategories, including linear path, angular path, average linear speed, average angular speed, instantaneous linear velocity, instantaneous angular velocity, average linear acceleration, average angular acceleration, instantaneous linear acceleration, and instantaneous angular acceleration. The models that utilized tip motion data (TM, TMV, TMG, TMVG) selected features from all ten subcategories. In contrast, Model TMVG used fewer features from each subcategory, with the largest number of features coming from the angular acceleration subcategory. Models TM, TMV, and TMG selected more features related to path length in each direction compared to Model TMVG, with Models TM and TMV selecting the most path-length-related features of the four. These three models also relied more heavily in general on individual features for each tool, whereas Model TMVG more heavily relied on features that took the ratio of values between the two tools.

### Best model features

Finally, we examined the features chosen for the top-performing Model TMVG to glean insights into which types of instrument movements may most closely relate to skill at the studied task. Model TMVG selected seven features in the linear path tip motion subcategory, including three features related to linear path in the y direction (in–out motion), two in the z direction (up–down motion), one in the x direction (left–right motion), and one for total path length. Five of these features represented ratios or summations of values from both tools, while the remaining two were related to Maryland movement in the y and z directions. Each direction of rotation was approximately evenly represented in the seven features selected from angular path, with rotation around the x-axis (tilting the tool up and down) being represented in three features. Similar to path length, five of these features represented ratios or summations of values from both tools, with the remaining two being related to Maryland rotation around the y-axis (approximately rotation about the shaft of the tool) and needle driver rotation around the x-axis (tilting the tool up and down).

Four features were chosen from the average linear velocity subcategory, with one feature related to each direction and the last feature related to the average speed overall. Of these four selected features, two gave ratios between the tools, and the remaining two were related to average velocity in the x and y directions for the Maryland. Four angular velocity features were also selected for the final model, and these features did not include any values related to the average rate of change of rotation around the x-axis (tilting the tool up and down). Rate of change of the angle around the y-axis (rotation approximately about the instrument shaft) was selected as an important feature for both the Maryland and the needle driver.

Features related to the instantaneous linear and angular velocity of each tool represented 12 and 11 features in the final TMVG model, respectively. The number of features related to movement in each direction was relatively equal in the case of instantaneous linear velocity, while most features chosen from the available instantaneous angular velocity options represented rotations around the z-axis (bending instruments left and right). In both cases, many features generated by peak analysis were chosen for the final model.

Only two features were selected from the average linear acceleration subcategory, including the ratio of the average acceleration in y for both tools and the ratio of the average acceleration in z for both tools. The only feature selected from the average angular acceleration subcategory was the ratio of the average accelerations about the y-axis (rotating approximately around the axis of the tool).

Of the ten tip motion subcategories, the instantaneous linear and angular acceleration categories contributed the most features to the final model. Instantaneous acceleration in the y direction was best represented in the linear acceleration subcategory, with seven features contributed; x and z linear accelerations contributed three features each. Angular accelerations about the x-axis and the y-axis contributed six features each to the final model, and angular accelerations about the tool z-axis contributed three.

Eight features related to grip motion were selected for the final model, including three features generated from peak analysis of Maryland velocities, the ratio of non-zero to zero velocity and acceleration in both tools, and one feature generated from peak analysis of needle driver acceleration. The last three features selected for the TMVG model were related to the visibility of the needle driver in the workspace.

## Discussion

The results of the study enable us to evaluate the strengths, weaknesses, and limitations of the proposed approach, as well as its potential utility in surgical training. The summed OSATS scores given by the blinded expert rater ranged from 5 to 20, yielding rounded averages from 1 to 4. Resident trials earned a wide variety of ratings, with rounded average scores ranging from 1 to 4, while all medical student trials received rounded average scores of 1 or 2 and all fellow trials received scores of 3 or 4. These results closely follow our expectations for performance at each training level and provide a level of validation to the scores given by our expert rater. Additionally, the variability of scores received by subjects in each group, especially the residents, shows that skill and level of training are not perfectly correlated.

Most of the 31 participants who completed two trials improved their summed score on the second try, as would be expected due to learning. The five subjects whose summed score decreased on the second attempt dropped at most 2 points, which is 12.5% of the 16-point range. For the most part, the differences between scores decreased with increasing first trial score, showing more consistent performance at the higher levels of skill. Again, these trends help validate our expert ratings and simultaneously underline the importance of evaluating a particular task performance rather than the trainee.

The six predictive models created from the motion data performed better on previously unseen trials than both the random and median prediction models with the exception of Model TM, which performed slightly worse than the median model when predicting summed score within 4 and rounded average score within 1. Models TMV and TMG both performed better than Model TM in all measures of accuracy, showing that the visibility and grip features added important information that was not provided by the tip motion data. This notion is further validated by the fact that the best performing model was Model TMVG, which utilized features from all four types of sensors.

We were especially surprised to find that Model TM exhibited the worst performance of the six tested models, as task completion time and tip motion are known to pertain closely to surgical skill. Upon further inspection, we found that this model had some of the highest accuracies and correlations on the training data but the largest decrease in performance from training to testing. These two facts lead us to believe that this model was overfitting to the training data, making it less generalizable for future trials.

Model TMVG was superior to all five of the other developed models when rating trials from a subject whose data were not used in training. In addition to having the highest accuracies and correlations, this model also had the best agreement between training and testing performance. This model was the only model of the six that predicted all rounded average scores within 1 point of the reviewer-generated scores. Additionally, this model was the most consistent in terms of correctly classifying each score, whereas other models correctly classified some scores with much more consistency than others. For example, Model T was able to correctly classify rounded average scores of 1 with an accuracy of 0.78, but it classified scores of 2 with an accuracy of only 0.19.

Gaining insight into the characteristics of movement that distinguish between different surgical skill levels was of particular interest to our group. Several previous studies [31–33] have used task completion time as a predictive feature when comparing novice and intermediate surgeons to experts. Interestingly, our best model did not select any features based solely on task completion time, which shows that completion time is not necessarily predictive when evaluating surgeons on a finer scale. Upon broad inspection of the features selected by each model, we found that more features of the Maryland motion were selected compared to the number of features calculated from needle driver motion. This emphasis is likely related to bimanual dexterity and handedness, since 31 of the 32 subjects were right handed, and they all held the Maryland in their left hand.

Broad inspection of the models also showed that the addition of visibility and/or grip features to the TM model decreased the number of tip motion features selected. Further, the TM model’s tip motion features mostly relate to the movements of the individual tools, whereas Models TMV, TMG, and TMVG mainly employ tip motion features that compare motion between the two tools. Adding information from the camera motion sensor and the grip angle sensors seems to elucidate which tip motion features are redundant and generally increase the generalizability of the TM model.

A more detailed inspection of the features chosen for the TMVG model reveals that the visibility of the needle driver in the camera view is important in evaluating skill, whereas Maryland visibility is not as predictive. This result is again likely related to handedness and bimanual dexterity, as the Maryland was held in the non-dominant hand of our mostly right-handed participants: less skilled surgeons move their non-dominant hand less than their dominant hand, causing the Maryland to stay in frame by default. The grip features selected included several values related to changes in the grip velocity and grip acceleration for the two tools. The feature that counts the number of extreme changes in needle driver grip angle acceleration divided by the total trial time was of particular interest, as trainees who received rounded average scores of 1 tended to move the needle driver grip abruptly compared to the more fluid movements of the surgeons with higher skill.

Model TMVG selected 77 tip motion features, which was the smallest number of tip motion features used by any of the four models that use tip motion data. Of the 10 subcategories of tip motion, instantaneous angular acceleration was the most well represented in the best model. Most of these features were related to rotations of the instrument around the shaft of the tool and up and down in the box. We speculate that this first type of feature was predictive because rotating around the tool shaft feels natural in the environment of the box trainer, perhaps causing less experienced surgeons to overuse this movement. On the other hand, rotating the tool up and down feels less natural and thus may be more challenging for novice surgeons. Other categories of features used in this model pertain to instrument acceleration in and out of the box, which probably relates to depth perception, and to various instrument velocities and path lengths, which we believe are correlated with economy of motion.

This study has a number of strengths compared to previous studies and standards of training. First, our dataset was labeled with blinded ratings of each task performance rather than broader assessments of the subject’s level of training or past experience. The variations in score between trainees at the same level and even within individual subjects provide validation of the wisdom of this method. Rating skill automatically through motion analysis is also much faster than having a human rate each performance. While a computer can analyze an entire motion recording in a matter of seconds, the time that a rater takes to review a performance is always on the order of the length of the trial, requiring a significant human resource cost and burden to senior physicians. Relatedly, ratings done by a computer are purely mathematical and based on computed differences between skill levels rather than subjectively based on an expert opinion. Further, unlike a human rater, a computer is not biased by presentation order or trainee identity, and it can rate each performance without getting bored and without directly comparing to the trial presented immediately before the current trial.

We were also able to expand the characterization of surgical skill to include information about tool visibility and grip angle. Adding visibility parameters added relatively little financial cost (about 500 USD for an additional sensor) compared to the total cost of the motion-tracking system (about 4000 USD) as well as minimal computational cost (calculation time: 0.33 out of 13.29 s). Similarly, adding flex sensors to the handles added little financial cost (about 50 USD for two sensors and associated circuitry) as well as minimal computational cost (calculation time: 0.57 out of 13.29 s). As discussed above, information from both of these sensor streams significantly improved skill prediction, so other researchers should consider incorporating such sensors into their setups.

Our approach to modeling surgical skill is both flexible and extendable due to the infinite number of features that can be created from the motion data. By extension, the ability of this model to adapt to different numbers and types of features opens the door to giving each subject more granular feedback about their performance than can typically be provided by human raters in dry-lab settings. Using LASSO to automatically select the features that correlate best with human ratings, we provide a framework for back-tracking to the finer motions and techniques that make up surgical skill, down to the millimeter or degree.

Despite these strengths, the design of our system is complex and includes a number of customized electrical and software components that would be difficult for non-engineers to replicate. Additionally, the subjects completed the suturing task on inanimate materials rather than in vivo; although these components closely mimic a pediatric abdomen, they differ from what a surgeon experiences in the operating room. Instrumenting the tools with magnetic motion sensors also required the removal of metal components from the environment of the box trainer to avoid interference with the signal from the transmitter. Relatedly, the sensors were attached externally on the tools, and despite the care taken to prevent these sensors from interfering with the task, some interference is inevitable due to the small size of the box trainer and its tools. Developing our models also required human ratings, which were time consuming and human resource intensive to acquire. Lastly, while feature selection was automated in our algorithm, feature creation was a human-resource-intensive manual task. While LASSO is able to choose the most mathematically relevant features out of a feature set, it is limited to the features that are entered as inputs, and better features may be possible.

A number of factors also limited the scope of our study. While a range of subjects from varying levels of training were recruited, we were limited to the population of surgeons at CHOP and the University of Pennsylvania Health System. Relatedly, the expert performance for this task was based on the technique employed by Dr. Thane Blinman, who is responsible for the MIS training program at CHOP. His technique was not validated by other expert surgeons prior to this study. Further limitations arose from the fact that there are a limited number of truly expert pediatric minimally invasive surgeons, and their time demands proved prohibitive for study participation, with a fixed number of fellows per year and a larger number of rotating residents at different training levels. Surgeons at another institution would likely employ slightly different techniques and might present a broader range of skills than the participants included in our study, who did not vary significantly in technique used. Furthermore, our model was unable to rate rounded average scores of 5 due to the lack of participation of any individuals at this level of performance, such as attending surgeons. An additional limitation arose from the fact that we focused our efforts on a single task due to the limited time that each subject had available to participate and the fact that machine learning methods benefit from large data sets. The study was also limited by the fact that trial scores had to be generated by a human reviewer. We tried to minimize the effect of subjectivity in the rater’s scores by summing the scores from five OSATS categories and taking the rounded average, but different raters may have given different scores. Further, despite the fact that videos were randomized, only the tools were visible in the videos, and the rater was blinded to the identity of the subject, presentation order bias may have impacted the scores given to many trials. For example, if the previous video showed a particularly poor performance and the next was a large improvement, the better video may receive a 4, whereas it would have received a 3 if rated on its own. Lastly, while we have laid a foundation for giving specific, motion-based feedback at the end of each trial, this capability has not yet been implemented.

The findings from this research have a number of implications for MIS training both within and outside of pediatrics. Using a system like ours provides methods by which surgical training programs may use less direct supervision to train surgeons outside of the operating room without hindering the learning process. By extension, a box trainer such as the one presented allows surgeons to receive feedback every time they practice a task, and it can use tools that are identical to those used in the operating room. Besides providing feedback more frequently, using a wide variety of motion-based features creates the possibility of giving trainees more detailed feedback that would allow them to correct finer errors in their motions; we are particularly excited about the possibility of moving away from giving only numerical scores from 1 to 5 and instead providing the trainee with quantitatively founded recommendations on how to improve skill. If implemented well, this approach could alleviate some of the burden of teaching finer surgical movements from senior physicians. Lastly, the use of our pediatric box trainer addresses the need to create training systems that better prepare training surgeons for the more ergonomically challenging environment of a pediatric abdomen.

Future applications of this approach are extensive. To increase the robustness of our system, our analysis techniques could be expanded to recognize equivalent expert techniques from several training programs and hospitals. This expansion could possibly be used to identify where a surgeon trained or which procedures he or she has practiced most. A natural extension from this study would compare the training progression for subjects who receive numerical ratings and standardized feedback from a human rater compared to the progression for those who get more detailed ratings and feedback from automatic algorithms associated with the box trainer. Additionally, a statistical analysis of significance can be done to further determine the precise way in which each of the selected features contributes to surgical skill. Beyond the capabilities of the current system, future applications could be to test the ergonomics and comfort of new laparoscopic tools compared to the current standard, as well as testing other clinically relevant laparoscopic tasks inside of the box. A more complex application would be to completely remove the human rater from the models and use unsupervised learning methods such as neural networks and deep learning to find the natural boundaries between surgical skill levels. Finally, a future application that could provide even more enhanced feedback is adapting the current algorithm to track the trainee’s performance in real time and give feedback throughout the procedure, rather than only at the end. Specifically, this application could include the detection of phenomena such as ineffective repetitive movements and non-dominant hand neglect, two habits of training surgeons that we believe may be corrected more easily in real time rather than retrospectively.

## Electronic supplementary material

Below is the link to the electronic supplementary material.

Supplementary material 1 (MOV 244540 KB)
